# Meiotic chromosome movement: what’s lamin got to do with it?

**DOI:** 10.1080/19491034.2019.1572413

**Published:** 2019-01-24

**Authors:** Dimitra Paouneskou, Verena Jantsch

**Affiliations:** Department of Chromosome Biology, Max F. Perutz Laboratories, University of Vienna, Vienna, Austria

**Keywords:** Meiosis, chromosome movement, LINC complex, lamina, *C. elegans*

## Abstract

Active meiotic chromosome movements are a universally conserved feature. They occur at the early stages of prophase of the first meiotic division and support the chromosome pairing process by (1) efficiently installing the synaptonemal complex between homologous chromosomes, (2) discouraging inadvertent chromosome interactions and (3) bringing homologous chromosomes into proximity. Chromosome movements are driven by forces in the cytoplasm, which are passed on to chromosome ends attached to the nuclear periphery by nuclear-membrane-spanning protein modules. In this extra view, we highlight our recent studies into the role of the nuclear lamina during this process to emphasize that it is a highly conserved structure in metazoans. The nuclear lamina forms a rigid proteinaceous network that underlies the inner nuclear membrane to provide stability to the nucleus. Misdemeanors of the nuclear lamina during meiosis has deleterious consequences for the viability and health of the offspring, highlighting the importance of a functional nuclear lamina during this cell cycle stage.

**Abbreviations:** DSB: DNA double strand break; LEM: LAP2, Emerin, MAN1; LINC: LInker of the Nucleoskeleton and Cytoskeleton; RPM: rapid prophase movement; SUN/KASH: Sad1p, UNC-84/Klarsicht, ANC-1, Syne Homology

Haploid gametes are produced as a consequence of meiotic cell divisions, and genetic diversity is generated through the assortment and exchange of parental chromosomes via the programmed induction of DNA double strand breaks (DSBs). DSB repair through homologous recombination creates a physical connection between the parental homologous chromosomes, which promotes their proper segregation. Chromosome segregation requires their side-by-side alignment, which is ultimately stabilized by establishment of the synaptonemal complex [1]. In all organisms examined so far, chromosomes engage in rapid prophase movements (RPMs) in the prophase of the first meiotic division, and these are crucial for the timely and successful accomplishment of meiosis, for review see [2,3]. Prophase I comprises the distinct stages leptonema, zygonema, pachynema, diplonema and diakinesis, where the movements are most pronounced in zygonema/early pachynema. RPMs support the chromosome pairing process by promoting efficient synaptonemal complex assembly between homologs. When homologous chromosomes become aligned side-by-side they need to be prevented from becoming interwoven or interlocked. RPMs initiation during the leptotene/zygotene stages is driven by forces originating in the cytoplasm, which are transmitted to chromosome ends located at the nuclear periphery.

The transmission of cytoplasmic forces to chromosome ends is mediated by the highly conserved SUN/KASH (Sad1p, UNC-84/Klarsicht, ANC-1, Syne Homology) bridge, which spans the nuclear envelope and is referred to as the LINC complex (LInker of the Nucleoskeleton and Cytoskeleton; for review, see [4,5]). In *Caenorhabditis elegans*, the LINC complex consists of SUN-1 and ZYG-12, located in the inner and outer nuclear membranes, respectively. The N-terminus of ZYG-12 extends into the cytoplasm and interacts with dynein, thereby connecting to the microtubule cytoskeleton, whereas the N-terminus of SUN-1 resides in the nucleus and cooperates with chromosome domains called pairing centers, which couple the nuclear envelope-associated chromosome end to the movement apparatus, for review see [6]. Disruption of the SUN/KASH bridge prevents chromosome movement and pairing [7–11]. Chromosome movement is required for synaptonemal complex assembly between homologous chromosomes, in absence of movement it assembles between chromosome regions without homology and chromosomes frequently fold-back on themselves [8,12]. Thus, the nuclear envelope has a major role in chromosome movement.

Lamins are an integral part of the nuclear periphery and nuclear envelope. They belong to the family of type-V intermediate filaments and comprise an N-terminal head domain, a coiled-coil central region, and a C-terminal tail domain [13,14]. Lamin dimers can polymerize to form higher-order filamentous structures, which can crosslink to form a rigid network [15–19]. The coil-coiled and the head and tail domains are responsible for the formation of lamin dimers and polymers respectively. Extensive studies during mitosis and interphase showed that the flexibility of the lamina network highly depends on phosphorylations. For instance, in vertebrates and flies phosphorylation of a conserved amino acid in the head domain promotes lamin depolymerization during mitosis, making the nuclear lamina more soluble and flexible, for review see [20].

The nuclear lamina underlies the inner nuclear membrane and interacts with chromatin and a variety of nuclear envelope components (e.g. SUN-1, nuclear pore proteins, and LEM (LAP2, Emerin, MAN1) domain proteins [21]). Besides its involvement in chromatin anchorage and regulating DNA replication, gene expression and heterochromatin formation, the dense lamina network provides mechanical stability to the nuclear envelope [13]. Lamins are classified as A-type or B-type according to their expression pattern and structural properties [22,23]. Vertebrates and flies have both A- and B-type lamins; in contrast, *C. elegans* has a single B-type lamin isoform, encoded by the *lmn-1* gene [24]. Yeasts, the simplest and most studied eukaryotic meiotic model systems, do not have a lamina at all. Likewise in plants, lamins are absent, however the lamin-like proteins (Little Nuclei Genes) perform analogous functions [25,26]. As the rigid lamina network resides between chromatin and the nuclear envelope, it would be well placed to inhibit chromosome movements and their connections with the nuclear envelope. In our recent study, we investigated whether and how the nuclear lamina is remodeled in the *C. elegans* germline, particularly during the leptotene/zygotene stages of prophase I, where RPMs take place [27].

We showed that upon meiotic entry the amount of peripheral lamin is reduced and it becomes more sensitive to detergent, thus rendering it more ‘soluble.’ The increased solubility depends primarily on the meiotic kinases CHK-2 and (to a lesser extent) PLK-2, since both *chk-2* and *plk-2* mutants have a more stable lamina network. Impairment of the chromosome domains that recruit those kinases also decreased lamin solubility. We also found that LMN-1 is phosphorylated on several sites in two clusters flanking its central rod domain. Phosphorylation at one of these sites (Ser32) is significantly upregulated upon meiotic entry and is dependent on *chk-2, plk-2*, and the chromosome domains that couple chromosome ends to the nuclear periphery. A *lmn-1* phospho-mutant ([*gfp::lmn-1^8A^*]), in which all the identified phosphorylated residues flanking the rod domain were substituted by non-phosphorylatable alanines, was more resistant to detergent than the wild type. In these mutants the lamina might become a mesh, that behaves very rigid. With this mutant, we also showed that a more rigid lamina network delays meiotic entry, decelerates chromosome movements, and delays chromosome pairing and DSB repair. In agreement with the hypothesis that a lack of phospho-modification would prevent de-crosslinking of the lamina, we found that nuclear lamina disassembly is delayed during the first embryonic division in the [*gfp::lmn-1^8A^*] mutant, Figure 1.

Surprisingly, these phenotypes do not have a strong impact on the viability of the mutant or on the timing of nuclear envelope breakdown in mitosis under laboratory conditions. However, we noticed increased germline apoptosis in those mutants with a more rigid lamina network. In fact, when the apoptotic machinery was deactivated in the [*gfp::lmn-1^8A^]; ced-3(n717)* mutant, there was a significant increase in embryonic lethality (roughly a quarter of the embryos die) and diakinesis oocytes with abnormal chromosome structures can be seen. Thus, we concluded that a more rigid lamina network causes stochastic chromosome damage in the prophase of meiosis I, and that the resultant compromised oocytes are efficiently culled by the apoptotic machinery.

In a previous study, we identified a meiotic surveillance system mediated by SUN-1 phosphorylation. If meiosis is dysfunctional, then SUN-1 phosphorylation persists to extend chromosome movement and delay meiotic progression, thus providing more time for error correction [28]. By reasoning that this self-correcting mechanism might also be at work in the lamina de-crosslinking mutants, we generated the [*gfp::lmn-1^8A^];[sun-1::gfp^10A^*] double mutant. Strikingly, we observed abnormal chromosome structures such as entanglements and chromosomal interlocks in pachytene, a phenotype that is never observed in the wild type or any of the single mutants. These structures persisted until diakinesis and were associated with a significant increase in offspring lethality, for a summary see Figure 1.

**Figure 1.**
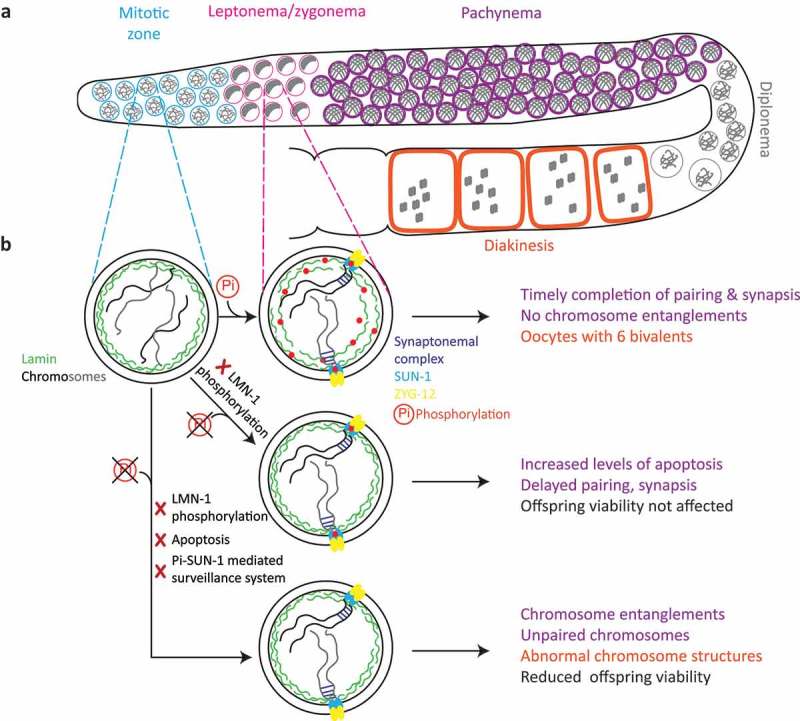
Impact of LMN-1 phosphorylation during meiosis in the *C. elegans* germline: (a) Schematic representation of meiotic progression along the *C. elegans* germline. Mitotic nuclei (blue) enter meiosis (pink), where the chromatin acquires a polarized configuration as a consequence of chromatin reorganization and chromosome movement. Nuclei then enter pachynema (purple) and homologous chromosomes are aligned in a side-by-side orientation. At diplonema (gray), chromatin condenses; during diakinesis (orange), oocytes with six bivalents can be seen in the wild type. (b) In interphase nuclei in the mitotic zone, non-phosphorylated lamin forms a rigid network. Upon meiotic entry, the lamina network is phosphorylated and adopts a more open structure. In leptonema/zygonema, chromosomes move, homologous chromosomes align, followed by assembly of the synaptonemal complex (blue lines) between homologs. Inhibition of phosphorylation renders the lamina network less detergent-soluble upon meiotic entry, which slows chromosome movement and delays pairing and synapsis. However, nuclei with abnormal chromosomes are culled by apoptosis during pachynema and overall offspring viability of the phospho-mutant is not affected. Deactivation of the apoptotic machinery or the phospho-SUN-1-mediated surveillance system combined with a more rigid lamina network leads to pachytene nuclei with chromosome entanglements and interlocks. Therefore, the resultant diakinesis oocytes have chromosomes with abnormal structures, which reduces offspring viability.

Link et al. (2018) highlight several important aspects of lamina network function in meiosis [1]: the mechanisms responsible for altering the physical properties of the lamina during nuclear envelope breakdown and for meiotic chromosome movement are functionally related [2]; the rigidity of the lamina network and the existing surveillance mechanisms in the germline are interdependent; and [3] lamina-modifying kinases are delivered and/or regulated from subtelomeric chromosome regions, near to chromosome end attachments to the nuclear periphery. These three points are discussed separately in the following text.

Regarding the first point, it is well established that phosphorylation/de-phosphorylation dynamically regulates the degree of rigidity of the nuclear lamina in mitosis and the solubility of lamin in interphase [20,29,30]. We propose that a similar mechanism exists during transition from mitosis to meiosis in the *C. elegans* germline. Phosphorylation and de-crosslinking of the lamina network on the nuclear rim coincides with the appearance of RPMs. When the lamina crosslinking (and thus its rigidity) is artificially maintained, as in the [*gfp::lmn-1^8A^*] mutant, chromosome movements are slower. By measuring the ability of chromosomes to diffuse along the nuclear envelope (by preventing force transmission through the cytoplasm), we confirmed that the more rigid lamina in [*gfp::lmn-1^8A^*] restricts chromosome end diffusion [27]. The failure to fully abrogate chromosome movement in the [*gfp::lmn-1^8A^*] mutant could be explained by the cytoskeletal forces being strong enough to overcome hindrance by the more rigid lamina. In addition, the more rigid lamina did not impair the interaction of chromosome ends with the nucleoplasmic component of the SUN/KASH bridge. Overall, the lamina is not eliminated, when RPMs take place, which supports the notion that the lamina itself does not impede chromosome end attachments. Lamin filaments are thinner than regular intermediate filaments [31] and obviously network density even in the mutant does not interfere with chromosome end attachments. We cannot exclude the possibility that the [*gfp::lmn-1^8A^*] mutant is a hypomorph, thereby having a less severe phenotype. However, we suspect that lamina rigidity is also mediated by many other lamina- and nuclear envelope-associated proteins, and therefore that lamina remodeling might be a multifactorial process. Although the lamina clearly persists for longer in mitotic cell divisions in the mutant [27], it is striking that lamina disassembly is not a rate-limiting step for nuclear envelope breakdown.

Regarding the second point of the existing surveillance mechanisms, it is clear that malsegregation of chromosomes causes aneuploidy, and can thus dramatically impair the offspring. Thus, mechanisms to prevent aneuploidy are in place at multiple levels during oogenesis. We could not identify the exact source of the observed chromosome abnormalities (such as ‘fused chromosome bodies’ or undefined chromatin masses in diakinesis): they might be caused by either delayed chromatin organization or less-efficient chromosome movement in the [*gfp::lmn-1^8A^*] mutant. In this study, however, we identified two mechanisms for preventing the segregation of fused/entangled chromosomes into oocytes: apoptosis and the prolongation of the movement process. In the ([*gfp::lmn-1^8A^];[sun-1::gfp^10A^*] mutant, preventing the self-correction mechanism of prolonging chromosome movement leads to chromosome interlocks in one-fifth of pachytene nuclei. Self-correction mechanisms have also been observed in *Sordaria*, where during zygonema chromosome interlocks appear in 20% of nuclei even in the wild type [32]. However, these structures are completely eliminated in pachynema, thus securing correct chromosome segregation. This study found that recombination factors were responsible for correct chromosome alignment, probably by resolving the entanglements. Recently, it was also reported in *A. thaliana* that impaired chromosome movement elicits chromosome interlocks that can be resolved through topoisomerase II-mediated topological rearrangements [33]. These examples emphasize that aberrantly connected chromosomes are efficiently eliminated at multiple levels to avoid their mis-segregation into oocytes.

Regarding the third point of the lamina-modifying kinases concentrated at the subtelomeric chromosome regions; experiments using the tractable genetic model, *C. elegans*, led to the identification of the homology recognition/pairing center regions, for review see [34]. These are subtelomeric regions (enriched in repetitive sequences) found on every chromosome, that couple the chromosome to the nuclear envelope while RPMs take place [35,36]. Pairing centers are necessary for the stable alignment and synapsis of homologous chromosomes, probably through their role in controlling chromosome movement [6,35]. They act as recruitment platforms for pairing center zinc-finger proteins, which then load polo kinase(s), a major regulator of chromosome movement [37–40]. Pairing center proteins accumulate at chromosome end attachments during RPMs; as the synaptonemal complex formation progresses, the polo kinase(s) relocalizes from pairing centers to the synaptonemal complex. In this tractable experimental model, the simultaneous deletion of pairing center proteins enabled the creation of a ‘pairing center minus’ genetic background [37]. Interestingly, both lamina solubilization and phospho-modifications depend on functional pairing centers; thus, pairing centers might be delivery or signaling platforms for kinases that modify the nuclear lamina. Therefore, pairing centers link chromosomal events, such as synaptonemal complex formation or meiotic recombination, with the nuclear periphery and in this way regulate RPMs by acting upon relevant substrates, such as lamin.

At first sight, the pairing centers seem to be an exotic worm-specific feature; however, they cooperate with highly conserved signaling kinases, such as CHK-2 and polo kinases. A role for those in other metazoans during chromosome movements has not been demonstrated to date. The discovery that these specific chromosome domains are involved in nuclear lamina modifications highlights the importance of studying such highly complex processes in relatively simple model organisms. It will therefore be interesting to investigate whether similar modules regulating a crosstalk between chromosome events and the nuclear lamina are also at work in higher eukaryotes.
